# Cost-Effectiveness of Cardiac Radiosurgery for Atrial Fibrillation: Implications for Reducing Health Care Morbidity, Utilization, and Costs

**DOI:** 10.7759/cureus.720

**Published:** 2016-08-01

**Authors:** Nikhilesh Bhatt, Mintu Turakhia, Thomas J. Fogarty

**Affiliations:** 1 Fogarty Institute for Innovation; 2 Department of Cardiology, Stanford University School of Medicine; 3 Fogarty Insitute for Innovation

**Keywords:** cyberheart, cardiac radiosurgery, atrial fibrillation, non-invasive, catheter ablation, cost effectiveness, utilization, radiotherapy, healthcare expenditures, cost

## Abstract

In the United States (U.S.), atrial fibrillation (AF) is the second-most common cardiovascular condition after hypertension, affecting four million Americans each year. Individuals with AF are three times more likely to be hospitalized over the span of a year when compared to medically matched control groups. The considerably large clinical population of individuals with AF mandates that the cost-effectiveness and efficacy of current treatment regimens for AF have egregious implications for health care spending and public health. Unfortunately, catheter ablation for AF treatment has been shown to make only modest gains in quality-adjusted life years, has yet to demonstrate cost-utility advantages over conventional therapies for AF, and has a reported rate of recurrence for AF that is notably high. Thus, there is a major unmet clinical need for a therapeutic option to treat AF that produces more consistent and efficacious results that are cost-effective. Cardiac radiosurgery as a therapy for AF has the potential to be remarkably cost-effective and produce robust patient outcomes. CyberHeart Inc. has developed the world’s first-ever cardiac radiosurgery (CRS) system designed to ablate the heart non-invasively. Procedures that ablate the heart utilizing the Cyberheart CRS system are anticipated to allow higher efficacy and more consistent results than current techniques such as catheter ablation. The aim of this study is to present the current healthcare utilization and expenditures in AF treatment, report the cost-effectiveness of catheter ablation for AF, and project the potential cost-effectiveness of cardiac radiosurgery for the treatment of AF.

## Introduction and background

Atrial fibrillation (AF) is an abnormal heart rhythm characterized by a rapid, disorganized, erratic electrical activation of the left and right atria, and upper chambers of the heart. Clinical manifestations may vary from common symptoms of rapid, irregular palpitations and exercise intolerance to more severe symptoms and signs of congestive heart failure [1-2]. In the United States, atrial fibrillation is the second-most common cardiovascular condition after hypertension, affecting four million Americans each year [3]. By the year 2030, AF is projected to affect more than 12.1 million Americans [3-6]. The prevalence of AF is six percent in Americans over age 65 (Medicare-eligible), and ten percent for those over age 80; 70% of individuals with AF are between ages 65 and 85 years [5, 7]. The absolute number of women and men with AF is equal, although above age 75, 60% of people with AF are women [5]. Currently, it is estimated that 15.8% of all patients diagnosed with AF belong to a younger clinical population (ages 20-59) [8]. However, due to rising rates of obesity and hypertension in young adults, the age-adjusted incidence of AF is increasing and is therefore expected to affect more Americans at a younger age [9].

Patients with symptomatic AF may experience palpitations, irregular beating, neck pulsations, non-anginal chest pain, extreme tachycardia, fatigue, exercise intolerance, sinus node dysfunction, and syncope. Failure to control rate or rhythm in symptomatic AF is associated with decreased quality of life, recurrent hospitalizations, anxiety and depression [10]. Additionally, AF doubles the risk of cardiovascular and all-cause mortality independent of anticoagulation status and AF risk factors [11-13]. AF may also cause or worsen heart failure due to left atrial mechanical dysfunction, irregular ventricular contraction, or sustained tachycardia from poor rate control [14].

The considerably large clinical population of individuals with AF mandates that the cost-effectiveness and efficacy of current treatment regimens for AF have egregious implications for health care spending and public health [15]. The aim of this study is to present the current healthcare utilization and expenditures in AF treatment, report the cost-effectiveness of catheter ablation for AF, and project the potential cost-effectiveness of cardiac radiosurgery for the treatment of AF. 

## Review

### Overview of current therapeutic options for AF treatment

Therapeutic regimens for AF treatment aim to prevent a thromboembolism and control symptoms [16]. The two management strategies currently utilized to treat AF can be classified as rate control and rhythm control [16]. While every situation of AF treatment is unique and requires a personalized management strategy, rate control treatment strategies are usually used when managing patients with asymptomatic AF, and rhythm control treatment strategies are typically used to manage patients with symptomatic AF [16]. Rate control treatment methods for AF attempt to control heart rate, do not require invasive procedures, and usually use drugs with low toxicity [17]. Conventional rate control strategies will consist of drugs such as beta-blockers, calcium channel blockers, or digoxin [4, 18]. For many patients, however, heart rate control is inadequate to improve the quality of life or ameliorate symptoms of heart failure. Rhythm control therapeutic regimens, the other strategy for AF management, attempt to restore sinus rhythm to the heart by using antiarrhythmic drugs (AADs) or invasive catheter ablation procedures [4, 17, 18]. Restoration of sinus rhythm is associated with improvement in physical functioning, general health, social functioning, and exercise performance [19]. Unfortunately, AAD therapy often entails high risks, can result in cardiovascular and non-cardiovascular toxicities, and is weakly effective, with AF recurrence rates reported being as high as 80% after one year [4, 17, 18].

Since 2000, there has been an exponential rise in the use of percutaneous radiofrequency catheter ablation to restore sinus rhythm in patients with AF. It must be noted however, that because the procedure is relatively new, the majority of practicing cardiac electrophysiologists did not receive formal training in AF ablation during the fellowship. Compared to other invasive electrophysiology (EP) procedures, left atrial ablation for fibrillation is more technically demanding, more expensive, and mandates more extensive operator training and cardiothoracic surgical backup. Unfortunately, left atrial-pulmonary vein isolation procedures for atrial fibrillation have been reported to have notably high rates of recurrence that have a variable range in the literature, with one to three-year freedom from recurrence ranging between 50-80% [20-23]. Although success rates display a small increase with second or third repeat catheter ablation procedure, doing so introduces significant risk and cost for treatment. However, it has been observed that among patients who do have treatment success, there is a marked improvement in the quality of life and even improvement of heart failure symptoms [24].

Complications of catheter ablation may be significantly higher than previous standards typically accepted for procedures in the modern era of medicine. In a recent study that examined the results of 4,200 AF ablation procedures from the California state hospital discharge database, 5% of patients had peri-procedural complications and 9% had hospitalization within 30 days [25]. The most common complications were vascular, relating to the catheter-based procedure. It was also observed that hospitals with low procedure volumes had the highest complications rates. Additionally, 22% of patients were hospitalized with AF within 12 months of ablation. Since a large portion of AF recurrences does not require hospitalization, the effectiveness of catheter ablation in clinical practice appears to be significantly lower than the 70-80% success rates from randomized trials performed at carefully-selected centers. Thus, the development of a procedure with more uniform efficacy and reduced complications remains a major unmet clinical need.

### AF healthcare utilization and expenditures

In 2001, 350,000 hospitalizations, five million office visits, and 276,000 emergency room visits were attributed to AF annually [26]. Medicare alone is estimated to spend $16 billion per year to treat newly diagnosed AF, most of which is spent on inpatient care to treat AF-related complications [27]. Estimates of the average annual healthcare system cost for patients with AF range from $20,613 to $40,169 [28]. Determinants that contribute to the high expenditures on AF management include the cost for visitations to outpatient clinics, hospitals, and emergency departments; laboratory tests, functional evaluations; and antithrombotic, anticoagulant, rate, and rhythm control drugs [29]. Of the total cost determinants, approximately 52-72% of the overall cost of AF treatment can be attributed to hospital [29, 30]. Furthermore, because AF patients often have other high-risk comorbidities such as heart failure, there are often further expenses required for vigilance for AAD therapy to prevent toxicities from occurring [29, 30].

Patients with AF have much greater health care utilization than patients without AF. A recent retrospective study conducted by Kim et al. analyzed a sample of approximately 90,000 patients with AF found that over a one-year span, patients with AF were three times more likely to be hospitalized than control subjects [31]. It was reported that AF patients were eight times more likely to have multiple cardiovascular hospitalization events and had more cardiovascular-related deaths than the control group [31]. Another retrospective study that analyzed the data from 58,555 patients displayed that hospitalization rates are three times more likely for patients with AF when there is a risk factor for diabetes, stroke, hypertension, or systemic embolism [29]. This surge in the likelihood of hospitalization corresponds with an increase in inpatient and outpatient care costs by an average of $6,988 and $4,541, respectively [29]. Similarly, it has been observed that among Medicare beneficiaries, in the first year after AF diagnosis, the patients with AF had a frequency of ≥ three hospital admissions (28% vs. 7%), ≥ three emergency room visits (14% vs. 3%), and ≥ three outpatient visits (72% vs. 61%) [32].

The study by Kim et al. also reported that the costs for patients with AF (n~90,000) are significantly greater than those of the medically matched control group of patients without AF [31]. For the AF group, the net incremental cost was $8705 when compared to the control group, with the total direct costs being $20,670 for AF group and $11,965 for the control group [31]. The largest discrepancy in costs between AF and control groups was inpatient services (average inpatient costs $7,841 and $2,622 for AF and control groups, respectively) [31]. The reported incremental cost of AF treatment of $8,705 per patient, which shows a 73% increase in expenditures for AF patients, was computed in 2008 and does not account for inflation. Other studies that had smaller sample sizes were noted to report higher incremental costs for AF patients of $12,349 (n=3944) and $14,199 (n=55,260) [31].

The current state of AF treatment indicates that AF is associated with high healthcare utilization and considerably steep costs. While the metrics reported in this study are from the United States (U.S.), AF is also a major driver of health-related expenditures worldwide, with EU expenditures estimated at €13.5 billion [29]. It has also been reported that the medical costs that can be attributed to undiagnosed AF (that eventually results in stroke sequelae or other conditions) are approximately $3.1 billion [33]. Furthermore, AF-associated cost and health care utilization has almost certainly increased as the prevalence of AF has risen over the last decade, and is anticipated to keep increasing in the future. 

### Cost-effectiveness of catheter ablation for AF

The success rates of AF ablation procedures depend on whether the patient has paroxysmal AF, persistent AF, and comorbidities such as heart failure [20-23]. Accordingly, the published literature that reports the rate of recurrence after initial catheter ablation for AF is highly variable, with one to three-year freedom from recurrence ranging from 50-80% [20-23]. A recent study that examined the outcomes of 328 patients with paroxysmal AF that received catheter ablation, found that after five years, only 59.4% of patients did not have the recurrence of AF after one procedure [4, 34]. The high rate of recurrence after undergoing the first ablation requires that second and third ablations for AF be performed to manage symptoms and restore sinus rhythm. While success rates were observed to have an improvement with repeat ablations when compared to initial ablations, it is also important to note that repeat ablation procedures lead to increases in risks for complication and results in higher overall costs of AF management [34].

The reported costs of catheter ablation for AF have been observed to have significant variations, even within the same country [35]. While hospital billing records report that the cost of catheter ablation for AF is $17,173 in the U.S., government reports indicate that the cost of catheter ablation in the U.S. is $26,584 [15, 35]. A recent study estimated that the cost for AF ablations that used CARTO/3D, non-CARTO/non-3D mapping, and NAVx systems had a range between $16,278 and $21,294; other studies reported costs of AF ablation ranging from $14,000 to $18,000 [28, 29]. Another study analyzed procedures of 26,000 patients in the US with commercial health insurance that underwent catheter ablation for AF from 2006 and 2011 [35]. It was found that the median cost was $21,300, and there was a 22% increase in cost from 2006 to 2011. The reported increase in cost was reported to be significantly greater than the expected increase due to inflation in the US over the examined time frame. Perino et al. also examined the cost variation and associated outcomes of catheter ablation for atrial fibrillation for 10,992 AF ablations [36]. It is possible that there is variation in costs of catheter ablation for AF because of re-hospitalization and repeat procedures due to recurrence. A recent study found that the one-year rate of hospitalization was 19.1%, and the one-year rate for repeat ablation was 16.1% [36]. The 30-day rate of procedural complications of perforation/tamponade was 3.8% [36]. Costs were stratified, and there was a small gradient in the improvement of outcomes, but there was also an increase in adverse events, which most likely was a driver of increased costs.

Unfortunately, catheter ablation has been shown to make only modest gains in quality-adjusted life years and has yet to demonstrate cost utility advantages over conventional therapies for a significant amount of patients. Cost-effectiveness analysis based on clinical trial data has shown an incremental cost-effectiveness ratio (ICER) of $51,000 per quality-adjusted-life-year gained [15]. However, when factoring in “real-world” effectiveness, utilization, and complication rates, cost-effectiveness is projected to be markedly worse and much higher than a willingness to pay thresholds in the United States. As a first-line therapy for paroxysmal atrial fibrillation, catheter ablation for atrial fibrillation did show benefit. In the ‘MANTRA-AF’ trial, a gain of an average of 0.06 quality life adjusted years (QALYs) to an incremental cost of €3,033, resulted in and an incremental cost-effectiveness ratio (ICER) of €3,434/QALY for patients younger than 50 years, and €1,08,937/QALY for patients with an age greater than 50 years [37]. Thus, radiofrequency (RF) ablation as a first-line strategy is a cost-effective treatment for younger patients. However for older individuals, and perhaps those with higher CHADS-Vasc scores there are not the same health economic benefits [28, 37]. Accordingly, it appears that the high cost-to-efficacy ratio of catheter ablation compromises the cost-effectiveness of the procedure.

Treatment that can prevent subsequent AF (and therefore its complications) would be expected to be highly cost-effective, as it would curb a patient’s trajectory of morbidity and health care expenditures. Over the last several years, increased attention has been devoted to improving procedural techniques with catheter ablation, along with improvements in ablation technology, such as routine use of intracardiac ultrasound, electroanatomical mapping systems, and anatomical ablation strategies. Although these tools and approaches are largely considered as the standard of care for AF ablation, they have not been shown to be associated with improvements in safety and effectiveness outside of single-center observational studies. Moreover, total procedure costs increases when these new techniques are used because these procedures require that additional disposable medical equipment to be used.

### Cost-Effectiveness of cardiac radiosurgery for AF treatment

### Clinical Rationale for Cardiac Radiosurgery for AF Treatment

It has been reported that there is a sizeable clinical population with AF and other comorbidities that are at a high risk for the current therapeutic options for AF treatment [4, 38]. Patients with AF and chronic kidney disease (CKD) are at a considerably high risk for stroke, and are, accordingly, placed on systematic anti-coagulants such as warfarin [4, 12, 39, 40]. However, it has been established that prescribing such patients with anticoagulants significantly increases the risk of hemorrhagic stroke and puts them at an unacceptably high risk of having a life-threatening bleeding event [4, 41-43]. Furthermore, patients with AF and CKD that undergo catheter ablation have unsatisfactorily high complication rates, with reports indicating complication rates for the procedure are 23.25% [4, 44]. The high complication rate for AF and CKD patients that undergo catheter ablation is believed to occur in large part because there is often vascular calcification observed for such patients, which increases the complexity of an already technically demanding procedure [4, 44].

CyberHeart Inc. has developed the world’s first-ever cardiac radiosurgery system designed to non-invasively ablate the heart. This novel technology precisely delivers radiation to cardiac targets with exceptionally high accuracy by utilizing the CyberKnife (Accuray, Inc., Sunnyvale, CA, USA) radiosurgical platform [4]. CyberHeart (CyberHeart Inc., Mountain View, CA, USA) procedures have a promising potential to provide efficacious therapy for AF patients that do not have acceptable clinical outcomes provided by the current treatment options. In addition to performing an entirely non-invasive ablation of the heart that will likely expand the treatable clinical population of individuals with AF, another advantage of the CyberHeart cardiac radiosurgery system (CRS) is that it allows the clinician to plan procedures using a completely anatomic approach and determine the exact location, size, and shape of the lesion before the procedure. Accordingly, the CyberHeart CRS is expected to decrease dependence on the technical competence of an electrophysiologist and produce more favorable outcomes with a reduction of complications that occur due to technical error. 

### Stereotactic radiosurgery is a cost-effective treatment strategy

From a cost-effectiveness standpoint, a significant benefit of utilizing stereotactic radiosurgery (SRS) to treat patients is that it enables is that SRS can be performed in an outpatient setting, thereby avoiding excessive costs associated with hospital admissions. A recent economic study performed a cost-effective analysis for using CyberKnife SRS to treat trigeminal neuralgia [45]. The two main therapeutic options compared in the report used to treat trigeminal neuralgia were microvascular decompression and SRS. In the population studied, both procedures were equally effective at six months, yet CyberKnife radiosurgery reduced hospital costs by an average of 34%. The robustness of this result was confirmed by sensitivity analysis [45]. In Germany, a study conducted by Bijlani et al. investigated the cost-effectiveness of SRS compared to surgical resection for acoustic neuroma, meningioma, and arteriovenous malformations [46, 47]. It was found that for patients who underwent microneurosurgery, the recorded average time required to recover in the intensive care unit (ICU) was 1.2 +/- 2.8 days and average total postoperative hospitalization time was 15.4 +/- 8.6 days. The significant amount of time that is required to be in a hospital for recovery was a principle reason for why the average cost per patient who underwent surgical treatment was higher (€15,252) than the average cost per patient who underwent SRS treatment (€7,920). Treatment with SRS instead of surgical resection was also observed to result in longer survival time and less healthcare utilization [46]. Banerjee et al. obtained similar results that displayed treating vestibular schwannoma with SRS, had a lower average cost per patient ($16,143) than treatment with microneurosurgery ($23,788) [46, 48].

CyberKnife SRS is also currently used to irradiate targets located on the body and has been shown to lead to superior clinical outcomes that are highly cost-effective [46]. Papatheofanis et al. recently performed a study that investigated the cost-effectiveness of CyberKnife SRS and external beam radiation therapy (EBRT) for the treatment of spinal malignancies [49]. The results of the cost-effectiveness analysis revealed that the patients who received treatment with CyberKnife SRS had an increase of .08 QALY and costs that were $1933 less than patients treated with EBRT [49]. Consequently, it was concluded that CyberKnife SRS therapy for metastatic spine tumors leads to improved clinical outcomes and is a cost-effective strategy. 

### Cost-effectiveness of CyberHeart cardiac radiosurgery for treatment of AF

It has now been established that catheter ablation for the treatment of AF has a high recurrence rate and does not have significant advantages in cost-effectiveness when compared other treatments for AF [4, 20, 23, 34]. Also, it has been presented that CyberKnife SRS used for the treatment of intracranial lesions and targets located on the body leads to improved patient outcomes and is highly cost-effective when compared to alternative treatment options. The principle reason why CyberKnife SRS is cost-effective is that it can be performed in an outpatient setting and, thus, removes costs usually associated with invasive procedures such as recovery time in the hospital and ICU. Accordingly, a therapeutic option for the treatment of AF that has comparable cost-effectiveness to that of CyberKnife SRS for the treatment of multiple conditions would be tremendously beneficial for the millions of people suffering from AF.

Cardiac radiosurgery as a therapy for AF has the potential to be remarkably cost-effective and produce efficacious patient outcomes at a lower cost than current treatment modalities. The proposed technology of CyberHeart has a projected Medicare reimbursement of $8,000, which is significantly lower than the Medicare reimbursement for catheter ablation ($12,500). Total costs for the episode of care are also anticipated to be sharply lower, since fewer ancillary studies and support, such as cardiac anesthesia, would be required for the CyberHeart procedure. Hypothetically, even if there were no improvement in effectiveness with CyberHeart compared to catheter ablation, there would still be substantial cost savings. Based on a conservative 5% improvement in effectiveness over catheter ablation, the cost-effectiveness estimations for CyberHeart’s technology are expected to improve further, especially when factoring in the reduction in re-hospitalizations that occur due to vascular complications with catheter ablation. Furthermore, because of the anticipated policy shift to cost bundling for 30 or 60-day episodes of care for common cardiovascular conditions, there may even be a greater incentive to select the CyberHeart technology over conventional catheter ablation to avoid the downstream excess in health care utilization and cost.

As cardiac radiosurgery becomes more mainstream, metrics that consider the differences in costs and the benefits of different interventions such as ICER will be used to decide its value and exact role as a therapeutic option [31]. Many governmental agencies have ICER thresholds for a cost-effective intervention [50]. Nonetheless, it is clear that the non-invasive therapy for AF treatment that the CyberHeart CRS system provides has a promising potential to change the dynamics of AF treatment by providing an efficacious therapy that is highly cost-effective. 

## Conclusions

Patients with AF have a multitude of cost determinants and are observed to have notably high health care utilization and expenditures. AF has been reported to account for 350,000 hospitalizations, 5 million office visits, and 276,000 emergency room visits over a one-year period, and is a major driver of healthcare costs in the U.S. and worldwide [26]. In the U.S. it is estimated that AF resulted in $16 billion in costs to Medicare alone for newly diagnosed patients and in the European Union (E.U). It has been approximated that expenditures on AF are more than €13.5 billion [27, 29]. Approximations of the average cost for a patient in the U.S. with AF per year range from $20,613 to $40,169 [28]. Individuals with AF are three times more likely to be hospitalized over the span of a year when compared to medically matched control groups [31]. Consequently, the cost-effectiveness and efficacy of AF treatment are tremendously important for the future of healthcare spending.

There is a major unmet clinical need for a therapeutic option to treat AF that produces more consistent and efficacious results, yielding lower complication rates, and allows treatment to be more cost-effective. Catheter ablation as a therapy to restore sinus rhythm to patients with AF has a reported rate of recurrence that ranges between 50-80% for one to three years after the initial procedure. The reported amount of patients that do not have AF recurrence after undergoing a catheter ablation after five years has been reported to be 59.4% [4, 20, 23, 34]. Because the greater majority of patients that have AF recurrence do not require hospitalization, it is likely that the already notably high recurrence rate of catheter ablation is even higher than the rate of recurrence reported in the literature. Such a high recurrence rate necessitates that repeat ablation procedures are performed to treat AF, which unfortunately have higher risks for complications and increase total costs for patients. Accordingly, studies on the cost-effectiveness of catheter ablation for AF have displayed that it is not economically beneficial or cost-effective as a first-line therapy for patients that are older than 50 years [28, 39].

Cardiac radiosurgery has a tremendous potential to provide therapy for AF that results in improved clinical outcomes that are more cost-effective than current treatment options for AF. CyberKnife SRS has already been established to be cost-effective for the treatment of intracranial neoplasms and cancer metastases located on the body [46, 47, 49]. CyberHeart CRS system is a cutting edge technology that delivers radiation to cardiac targets with impeccable accuracy to non-invasively ablate the heart. Because its proprietary technology allows clinicians to use an anatomic approach and predetermine the exact size and shape of the cardiac ablation on a computer program, procedures that ablate the heart utilizing the Cyberheart CRS system are anticipated to allow higher efficacy and more consistent results than current techniques such as catheter ablation. In addition to providing improved clinical outcomes, CyberHeart procedures for the treatment of AF are projected to be less expensive than catheter ablation procedures. A CyberHeart procedure for AF has an expected Medicare reimbursement of $8,000, which is comparably lower than the reported $12,500 Medicare reimbursement of catheter ablation for AF. The anticipated decrease in costs for a CyberHeart procedure for AF occurs because CRS does not require cardiac anesthesia, cardiothoracic surgical backup, or other support that is required for catheter ablation. While current research is ongoing to further validate the efficacy of the CyberHeart CRS system in an FDA approved clinical investigation, the benefits of non-invasively ablating the heart with CRS to treat AF in a cost-effective manner will undoubtedly have incredible implications that can radically improve the lives of the millions of individuals that suffer from symptomatic AF. 
